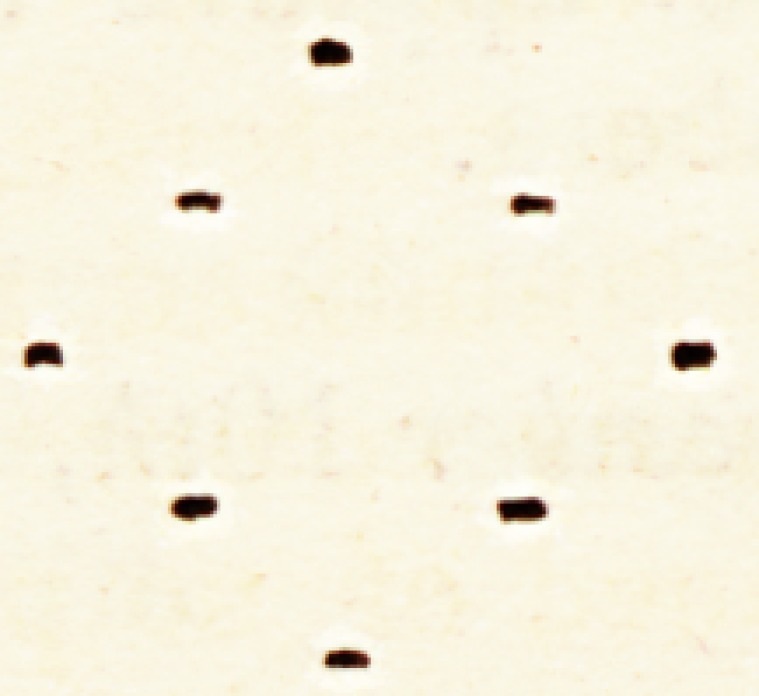# Intelligence

**Published:** 1827-01

**Authors:** 


### INTELLIGENCE.

### MONTHLY REPORT OF PREVALENT DISEASES.

During the past month the weather has been more than usually capricious; we have had some days of rather severe cold, but upon the whole a close, damp, muggy, and comparatively warm state of atmosphere has prevailed. Fever, which we have noticed in our last Reports as rather alarmingly prevalent, seems disposed to give way without constituting a regular epidemic; at least fewer cases of this nature have fallen under our observation during the past month than during either of the two which immediately preceded it. The sharp weather above alluded to gave rise to a considerable number of pleurisies, and other attacks of an acute inflammatory character; so that the lancet and tartarized antimony were for a short time in requisition about the end of November. No sooner, however, had the dull and wet weather taken place of the few days frost, than there was a recurrence of those abdominal derangements which constituted so large a portion of the diseases prevalent during the summer. These bowel complaints have been much more limited in number, but some of them quite as severe in degree, as those which occurred at an earlier period of the season. To the same oppressive state of atmosphere, and to the sedentary habits it almost necessarily produces, may, in part at least, be attributed the unusual number of nervous affections which have been recently witnessed by the writer. Under this general appellation of *nervous* we include hysteria, and all the cousins german of that numerous and widely connected family. In one instance, a young woman remained for three days in a state of insensibility, with dilated pupils, and other symytoms of a character *apparently* so well marked, as to render it difficult, or perhaps impossible, to distinguish it from true apoplexy, otherwise than by the previous and subsequent histories of the case, which unequivocally pointed out its hysterical nature.

*December* 25*th*.

The following note refers to the case of R. Fitcomb, given at page 11 of the present Number:—

My dear Sir,—I am sorry to be obliged to add a further report of the case of compound fracture which I amputated on Saturday last, which I will thank you to subjoin to my paper in any form you may think proper. On Monday night he became delirious, with a very rapid pulse, dry tongue, and hot skin; he did not complain of any pain in the stump, and there was not the least appearance of inflammation up the limb. When I visited him yesterday, his eye was deeply tinged with bile, and his whole skin had a subjaundiced appearance. His pulse was very frequent and sharp, but weak, and easily compressed; his tongue clean, but dry, and rather red at the tip. He complained of no pain. On dressing the stump, the edges were in close contact, and appeared to have united in many places: on pressing it lightly, a small quantity of unhealthy pus escaped near the centre. Conceiving that the constitutional irritation might be connected with this, I directed the stump to be poulticed, to favour the escape of matter; some calomel and opium to be taken at night; the head to be shaved, and bathed with spirit-wash; and a large blister to be applied to the nape of the neck. Towards evening he became violently delirious, and continued so until the following morning, when he sunk gradually, and died about 11 A.M. Permission could not be obtained to examine the body; but I have little doubt there was visceral disease, as I am informed that he had been for years in the daily habit of drinking twelve or thirteen glasses of undiluted spirits, besides much porter and ale. On examining the limb after amputation, a large loose portion of dead bone was found immediately between the fractured ends, which effectually prevented any attempt at reparation. The spine of the tibia was bare for some inches above the fracture, and from the groove commenced by the absorbents, there is no doubt that it had perished to this extent. The limb was minutely injected with size and vermilion, to shew the granulations, and any reparative process.

Dear Sir, very truly yours,

HENRY EARLE.

*George-street, Wednesday Night; Dec.* 13, 1826.

---

*Application of Lunar Caustic.*

Dear Sir,—I have just received a paper from Mr. HIGGINBOTTOM, containing "Directions for the Application of the Lunar Caustic." It is of course too late for the next Number of your Journal, but I thought you would have the kindness to announce the reception of the communication, *and call the attention of your readers to it,* observing that this remedy is followed by complete success or complete failure, according as it is applied properly or carelessly.

Yours very truly,

MARSHALL HALL.

15, *Keppel-street, Russell-square; Dec.* 19, 1826.

---

*Vaccination.*—The following observations on Vaccination, with reference to Dr. GREGORY's paper in our November Number, are from the pen of Mr. NORTH.

"In the performance of this very simple but most important operation, it is at all times highly desirable, and in many cases an object of the first importance to *ensure* its success."—I am indebted to Dr. Gregory* for this sentence, and willingly admit the truth it inculcates. But, as I am inclined to believe that some of the remarks which have been made by that gentleman on vaccination are more likely to lead to a *failure* of the operation than to ensure its success, I am induced to state my opinion very briefly upon the subject.

The use of a very sharp lancet is urgently recommended by Dr. Gregory for the performance of vaccination. He observes, "it has frequently occurred to him to notice that a lancet which has been successfully employed in venesection, is yet not sufficiently sharp for the purposes of vaccination." I will not venture to say that Dr. Gregory is singular in this opinion, but I know that most practitioners prefer a lancet with a roundish and rather blunt point. A very sharp lancet is objectionable from the flow of blood it causes, by which the lymph is either washed out of the puncture, or so diluted as frequently to render the operation unsuccessful. An instrument of the former description may be employed, and produce scarcely an appearance of blood.

The next point upon which I shall venture to offer a few words, appears to me of much importance. Dr. Gregory states "that the most complete effect, both upon the arm and constitution, is made by *six* or *eight* punctures, supposing them all to be effectual." We are directed to make them "in a circular form, and at moderate distances; as thus —

The advantage of the circular form is, that the true figure of the areola is thus preserved." But I must be allowed to suggest that there is no advantage in ensuring the formation of an areola in this manner. It is clear that from each puncture a certain extent of inflammation may arise, and that the united inflammation of "the six or eight" may form an areola, although no single puncture might have produced this appearance. The guide upon which so much stress has been laid is the production of an areola from a *single* puncture, and consequently it is desirable, if several punctures be inserted, that there should be

* Remarks on the Practice of Vaccination, by Dr. GREGORY. (London Medical and Physical Journal, November 1826.) p. 410 - vol IV.

space enough between them to prevent the spread of inflammation from one to the other, that the criterion may not be destroyed.

Provided the other directions are complied with, Dr. Gregory is perfectly indifferent " whether little or much blood flows from the wounds."—Here again we are at issue. From my own observation I am induced to believe that, the less the flow of blood, the greater the probability of success; and I am much deceived if this is not the general opinion of the profession. In a copy of Dr. Jenner's work on Vaccination, I find a manuscript note, in the hand-writing of Dr. Bateman, from a paper of Dr. G. Fordyce on Variolous Inoculation, which is exactly in point, as it bears with equal force on vaccination. "I apprehend (says Dr. Fordyce) that the *principal*, if not the *only*, consideration in inoculation, is *the manner of making the puncture*, which should *penetrate the scarf skin*, so that it may be felt on raising the point of the lancet: *if no blood appears, the better*." It would be very easy to accumulate evidence in support of this opinion, if it were necessary.

Dr. Gregory objects to more than six or seven subjects being vaccinated " from even a very tumid eighth-day vesicle."—I believe there can be no impropriety in vaccinating many more, if there should be any scarcity of matter. He also informs us, that "it is obvious that, the younger the lymph, (fourth or fifth day,) the greater is its degree of intensity." I doubt this fact as applied to the vaccine lymph. If we were to search for analogies from the perfect development and formation of other morbid poisons, we should find many reasons to suppose the dogma of Dr. G. erroneous. That he is also opposed to the general opinion upon this subject, may be presumed from the eighth-day vesicle being usually preferred; and upon this point, indeed, Dr. G. appears to be *impar sibi*; for, in his recommendations to young vaccinators, he directs them to take lymph "of the sixth, seventh, or eighth day," when, according to his own doctrine, it is no longer at " its greatest degree of intensity."

Dr. Gregory states that the degree of local inflammation and general distress will not be increased by the number of punctures he recommends. In several cases, however, I have seen very severe and unmanageable inflammation, and great general disturbance, from the practice of making numerous punctures. I believe such instances are by no means common; but as troublesome symptoms rarely, if ever, occur, either locally or generally, from the insertion of two or three punctures made at proper distances from each other, and as no greater degree of security is conferred by increasing that number, I should never adopt the plan recommended, as it exposes the child (particularly if it be of an irritable constitution) to an unnecessary risk. I believe it to be the same with respect to the vaccine as the variolous disease: the latter is as completely produced by one puncture as by many. But, to keep up the necessary supply of vaccine lymph, and to render the success of the operation more certain, as one puncture may entirely fail, two or three are usually made. If but one puncture is applied, it ought not to be disturbed.

If the observations on Vaccination had proceeded from a less respectable source, I should not have replied to them: but the opinions of Dr. Gregory, particularly upon a subject to which he has devoted so much of his attention, ought not to pass by unnoticed, as they will be considered very safe guides by, at least, the junior members of the profession.

To offer any apology to Dr. Gregory for having thus unreservedly commented upon his doctrines, would be to imply the unjust suspicion that he is not actuated by that zeal for his profession, which must rather approve than deprecate the utmost freedom of discussion amongst all its members.

J. NORTH.

*Upper Berkeley-street, Portman-square; November 10th*, 1826.

---

*Necrology*.—Some of the most distinguished ornaments of our profession, both at home and abroad, have died during the last few months.

Laennec, so well known for his application of mediate auscultation to the diagnosis of diseases of the chest, died in September. Although we are among those who think the value of the stethoscope is at present very greatly overrated by some, still we are ready to do justice to the talents and industry of M. Laennec, and to the great mass of pathological information he had acquired. It is to the practical knowledge acquired by many years of industrious research, *without the stethescope*, that in our opinion may be attributed the reputation which he afterwards gave to this instrument,—many ascribing to it the result of thirty years' patient investigation and laborious study.

LAUTH, Professor at Strasbourg, known as the author of various anatomical and other works, is lately deceased.

VACCA BERLINGHIERI, the distinguished Professor of Pisa, likewise died in September; and SCARPA, the father of Italian surgery, has terminated his long and brilliant career.

To this list of distinguished men, we have to add the name of Dr. BARCLAY, of Edinburgh. He had been a lecturer on anatomy for about thirty years, and his learning and industry were shewn by various professional works, and by the collection of an extensive and valuable Museum. He died on the 21st of August, in his sixty-sixth year.

---

*Animal Magnetism.*—The members of the profession in this country will be amused to learn, that the Academie de Medecine of Paris has very recently established "*une Commission permanente, pour s'occuper du Magnetisme Animal,*" Credat Judæus!

---

*Literary Notice.*—Dr. REECE has in the press, An Examination, Chemical, Physiological, and Therapeutical, of Dr. King's Pamphlet, entitled "Observations on the Artificial Mineral Waters prepared by Dr. Struve at Brighton," with practical remarks on the medicinal virtues of the waters, and an analysis of the cases in which it is said they have proved beneficial.

### METEOROLOGICAL JOURNAL,

*From November 20th, to December 20th,* 1826.

By Messrs. HARRIS and Co. Mathematical Instrument Makers, 50, High Holborn.

| November | Moon. | Rain gauge. | Thermom. | | | Barometer. | | De Luc's Hygrom. | | Winds. | | Atmospheric Variations. | | |
|---|---|---|---|---|---|---|---|---|---|---|---|---|---|---|
| | | | 9 A.M. | MAX. | MIN. | 9 A.M. | 10P.M. | 9 A.M. | 10P.M. | 9 A.M. | 10P.M. | 9 *a.m.* | 2 *p.m.* | 10 *p.m.* |
| 20 | | | 44 | 47 | 42 | 30.24 | 30.33 | 85 | 93 | NE | NE | Cloud | Cloudy | Cloudy |
| 21 | | | 45 | 46 | 40 | 30.35 | 30.33 | 75 | 86 | NE | NNE | — | — | — |
| 22 | ☾ | | 44 | 46 | 43 | 30.27 | 30.17 | 77 | 81 | N | N | — | — | — |
| 23 | | | 44 | 49 | 43 | 29.97 | 29.76 | 93 | 88 | NNE | NE | — | — | Sm. Ra. |
| 24 | | | 45 | 47 | 32 | 29.41 | 29.31 | 90 | 80 | WSW | WSW | — | — | Fine |
| 25 | | | 38 | 42 | 28 | 29 08 | 29.11 | 74 | 89 | WSW | WSW | Fine | — | Cloudy |
| 26 | | | 31 | 36 | 30 | 29.21 | 29.40 | 75 | 83 | WSW | W | — | Fine | Foggy |
| 27 | | | 33 | 37 | 32 | 29.60 | 29 64 | 83 | 88 | W | SW | — | — | — |
| 28 | | | 35 | 48 | 47 | 29.68 | 29.43 | 91 | 96 | WSW | SW | Foggy | Sm. Ra. | Sm. Ra. |
| 29 | ● | | 48 | 50 | 43 | 29.28 | 29.31 | 87 | 93 | SW | SW | — | Fair | Rain |
| 30 | | | 46 | 48 | 36 | 29.28 | 29.40 | 94 | 94 | WSW | W | Cloudy | — | Foggy |
| Dec. | | | | | | | | | | | | | | |
| 1 | | | 45 | 46 | 39 | 29.33 | 29.13 | 93 | 97 | WSW | WSW | — | Rain | Rain |
| 2 | | | 42 | 47 | 39 | 29.10 | 29.10 | 87 | 87 | WSW | W | Fair | — | Fair |
| 3 | | | 40 | 45 | 34 | 29.29 | 29.31 | 84 | 85 | W | W | Cloudy | Cloudy | Cloudy |
| 4 | | | 37 | 39 | 35 | 29.31 | 29.61 | 88 | 82 | WNW | NW | — | — | — |
| 5 | | | 40 | 41 | 33 | 29.62 | 29.56 | 84 | 89 | NW | NW | — | — | Rain |
| 6 | ☽ | | 36 | 50 | 50 | 29.60 | 29.62 | 98 | 98 | ESE | SSW | Rain | Rain | — |
| 7 | | | 52 | 54 | 47 | 29.50 | 29.33 | 99 | 98 | WsW | SW | — | — | Cloudy |
| 8 | | | 50 | 52 | 42 | 29.20 | 29.45 | 90 | 88 | SW | W | Cloudy | Cloudy | — |
| 9 | | | 42 | 48 | 47 | 29.76 | 29.73 | 92 | 97 | W | S | — | — | Rain |
| 10 | | | 52 | 54 | 49 | 29.74 | 29.80 | 99 | 97 | SSW | S | Rain | Rain | Cloudy |
| 11 | | | 52 | 53 | 46 | 29.73 | 29.80 | 98 | 95 | SSW | SSW | — | Fair | Fair |
| 12 | | | 50 | 51 | 44 | 29.71 | 29.46 | 95 | 94 | S | SSE | Fair | — | Cloudy |
| 13 | | | 48 | 52 | 43 | 29.48 | 29.49 | 97 | 97 | SW | SW | — | — | — |
| 14 | ○ | | 46 | 50 | 45 | 29.49 | 29.52 | 91 | 92 | SW | S | — | — | — |
| 15 | | | 47 | 49 | 45 | 29.53 | 29.50 | 90 | 97 | SE | E | Foggy | — | — |
| 16 | | | 47 | 49 | 44 | 29.47 | 29.60 | 98 | 97 | E | E | Cloudy | — | — |
| 17 | | | 45 | 46 | 41 | 29.75 | 29.84 | 98 | 94 | E | ENE | — | — | Fair |
| 18 | | | 42 | 43 | 40 | 29.87 | 29.92 | 89 | 85 | E | E | — | — | — |
| 19 | | | 42 | 44 | 40 | 29.92 | 29.94 | 87 | 90 | E | ENE | — | Cloudy | Cloudy |

The Rain-gauge having frozen, no account was taken of the quantity of Rain fallen.

## Figures and Tables

**Figure f1:**